# Hiding in Plain Sight: A Case for Cryptic Metapopulations in Brook Trout (*Salvelinus fontinalis*)

**DOI:** 10.1371/journal.pone.0146295

**Published:** 2016-01-05

**Authors:** David C. Kazyak, Robert H. Hilderbrand, Tim L. King, Stephen R. Keller, Vikram E. Chhatre

**Affiliations:** 1 University of Maryland Center for Environmental Science, Appalachian Laboratory, Frostburg, Maryland, United States of America; 2 U.S. Geological Survey, Leetown Science Center, Aquatic Ecology Branch, Kearneysville, West Virginia, United States of America; 3 University of Vermont, Department of Plant Biology, Burlington, Vermont, United States of America; Bournemouth University, UNITED KINGDOM

## Abstract

A fundamental issue in the management and conservation of biodiversity is how to define a population. Spatially contiguous fish occupying a stream network have often been considered to represent a single, homogenous population. However, they may also represent multiple discrete populations, a single population with genetic isolation-by-distance, or a metapopulation. We used microsatellite DNA and a large-scale mark-recapture study to assess population structure in a spatially contiguous sample of Brook Trout (*Salvelinus fontinalis*), a species of conservation concern. We found evidence for limited genetic exchange across small spatial scales and in the absence of barriers to physical movement. Mark-recapture and stationary passive integrated transponder antenna records demonstrated that fish from two tributaries very seldom moved into the opposite tributary, but movements between the tributaries and mainstem were more common. Using Bayesian genetic clustering, we identified two genetic groups that exhibited significantly different growth rates over three years of study, yet survival rates were very similar. Our study highlights the importance of considering the possibility of multiple genetically distinct populations occurring within spatially contiguous habitats, and suggests the existence of a cryptic metapopulation: a spatially continuous distribution of organisms exhibiting metapopulation-like behaviors.

## Introduction

The management and conservation of genetically distinct groups of organisms is a central pillar of modern conservation biology. However, defining populations can be an important challenge for understanding basic natural history as well as for recognizing appropriate units of biodiversity for conservation and management. Many populations show local adaptation to their environment, which may be expressed as variation among populations in life history strategies [1], physiology [2], and morphology [3]. Reproductively-isolated populations can even develop in sympatry, sometimes as the result of divergent selection pressures (e.g. [4], [5]). Genetic differences among proximate populations may reflect a history of local adaptation and provide a reservoir for potential adaptation to changing conditions in the future.

An understanding of population structure is important for effective management. Inventories of genetic diversity and population size may be misleading if population structure is not adequately understood [6]. Similarly, groups of populations can exhibit a portfolio effect, where variation in overall abundance is dampened by the independent trajectories of individual populations, thus increasing overall resilience but masking the dynamics of individual populations [7]. In both scenarios, our understanding of the status and trends of populations may be obscured if the scale of our observations is not appropriate to the underlying biological system. Furthermore, restoration activities may have unexpected results if they do not consider patterns of connectivity and local adaptation [8]. Consequently, understanding the structure and boundaries of populations is necessary to implement effective management strategies.

Spatial heterogeneity and connectivity among suitable habitats can substantially influence both the structure and the boundaries of a population. While outright barriers to dispersal bound populations, biotic and abiotic factors may restrict connectivity between groups of individuals [9], [10], and lead to patterns of population isolation by ecological features of the landscape rather than purely by geographic distance (e.g. isolation by resistance [11]). In terrestrial environments, organisms can usually disperse in many directions across the landscape, and most landscape features change slowly through time. In contrast, streams are highly dynamic linear environments with limited connectivity between adjacent watersheds [9], [12], [13]. Under these conditions, heterogeneity in stream networks is expected to contribute strongly to population structure [14].

Despite the differences between streams and terrestrial environments, individuals that occupy spatially contiguous habitat within a stream network are typically assumed to represent a single population. This assumption is important, pervasive, and generally untested. Conceptually, there are several alternative scenarios to a randomly mating (e.g., panmictic) population that need to be considered. A single population may exhibit genetic isolation by distance [15], [16], where gene flow is spatially restricted. In contrast, multiple geographically discrete and reproductively isolated populations may be present [17], [18]. Finally, fish within a stream network may represent admixtures of genotypes originating from different local subpopulations within a regional metapopulation [19]. Such alternative population structures may be widespread yet overlooked because of the linear nature of stream networks. In many cases, they can only be detected with molecular techniques.

These alternative population structures within a stream network require different approaches to management. For example, groups of populations may exhibit a portfolio effect, potentially obscuring the declines of individual populations [7], and calling for population-specific management or policies which broadly protect the fishery resources. Even within a population, different life history strategies may have different geographic distributions. For migratory fishes, this may result in aggregations of migratory individuals from different populations during non-reproductive periods, as has been observed in numerous salmonids [19], [20]. Furthermore, if local adaptation has occurred, populations could differ in key measurable traits with impacts to our understanding of population dynamics. These scenarios highlight the importance of characterizing the structure of populations so that we may more effectively understand a species and its surrounding landscape.

Brook Trout (*Salvelinus fontinalis*) are a broadly distributed and well-studied species that exhibits considerable life history variation [21–23], and genetic diversity [24], [25]. Widespread declines have attracted attention, and millions of dollars are spent annually on the conservation and restoration of Brook Trout. These management activities have frequently been met with unexpected results (e.g. [26]), potentially as the result of misunderstanding population structure and function.

Our ultimate goal was to explore the population structure of stream resident Brook Trout in a highly connected stream network in hopes of better understanding its characteristics with respect to management. In this paper we test the hypothesis that Brook Trout in a connected stream network represent a single, panmictic population against the alternatives involving population differentiation on small spatial scales. Where differences existed, we further explored relationships in key measurable traits.

## Materials and Methods

**Fig 1:**
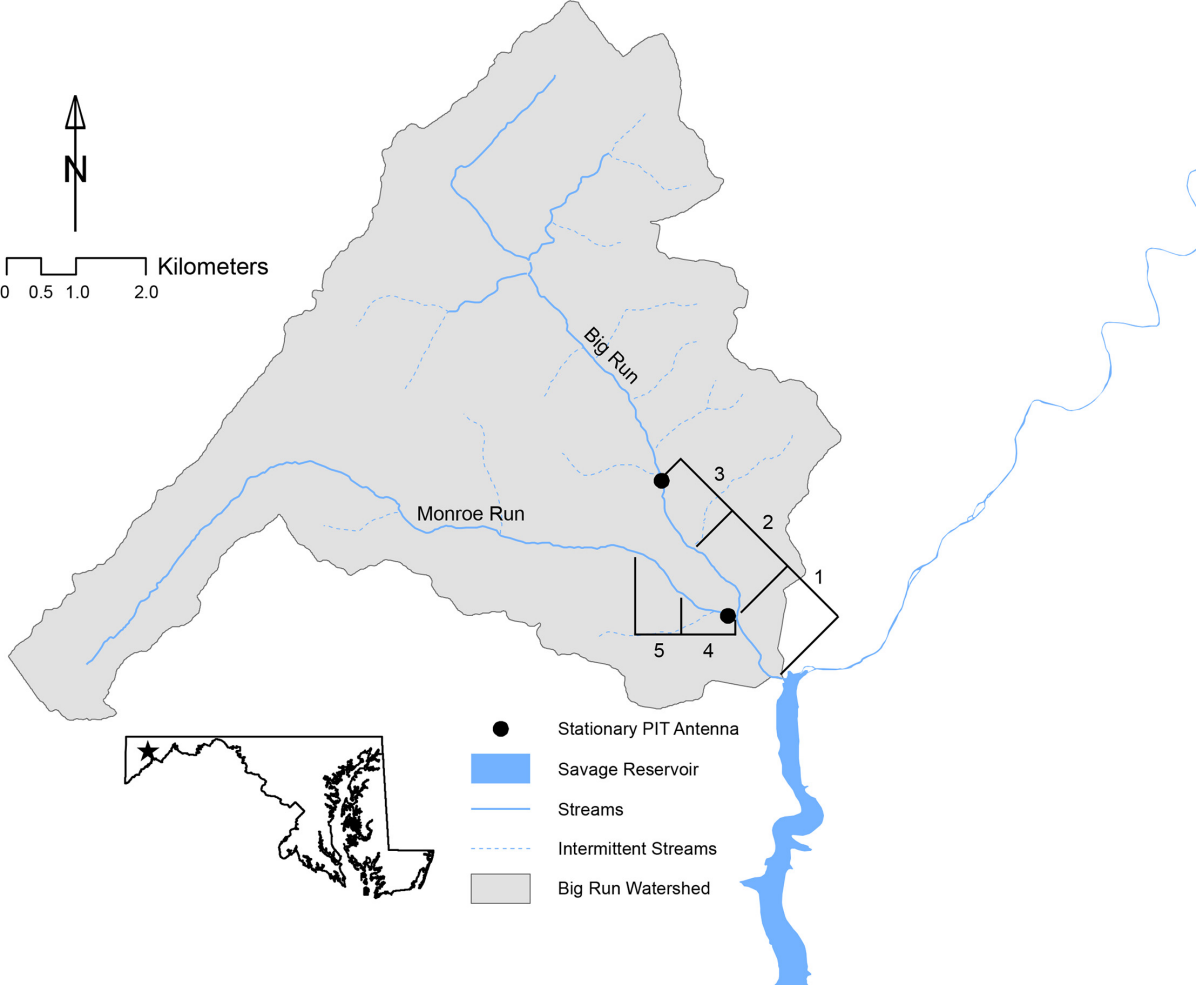
Map of the study area. We focused on five contiguous sections of the Big Run watershed in western Maryland. Stationary passive integrated transponder (PIT) antennas were operated at two sites within the study area.

The Savage River watershed of western Maryland contains >100 km of interconnected Brook Trout habitats [27] and has been identified as a regionally important population stronghold. Within this larger drainage, we focused on the Big Run watershed, which contains about 24 km of perennial streams supporting Brook Trout. The Big Run watershed contains one major tributary named Monroe Run, which forms a Y-shaped network (Fig 1). The study streams support a typical Appalachian Plateau stream community dominated by Brook Trout, Blue Ridge Sculpin (*Cottus caeruleomentum*), and Eastern Blacknose Dace (*Rhinichthys atratulus*). We concentrated our efforts on 4.5 km of stream closest to the downstream terminus with the upper Savage River.

Brook Trout were collected biannually (2010–2013) in the summer (June-July) and autumn (September) using backpack electrofishing across the entire study area. Following capture, all individuals were chemically anesthetized (80 mg/L tricaine methanesulfonate buffered with 0.2 mM NaHCO_3_, pH = 7), measured, and scanned for a passive integrated transponder (PIT) tag (see S1 Dataset for capture records). During summer sampling, any previously un-captured individuals were implanted with 12 mm PIT tags. Adipose fin biopsies were obtained from each individual and preserved in 95% ethanol for genetics analysis. The field sampling described in this manuscript was approved by the University of Maryland Center for Environmental Science Institutional Animal Care and Use Committee (approvals F-AL-08-08 and F-AL-11-03).

### Population Genetics

We selected 250 Brook Trout from across the study area for genetic analysis. Individuals were chosen using a stratified random sampling design where about 50 individuals were selected from each of five stream reaches, representing a mixture of stationary and mobile individuals. An individual was considered to be mobile if it moved >0.1 km between physical recapture events. The age of sampled Brook Trout was unknown, but it is highly likely that multiple generations were represented since we genotyped a broad size range of fish (70–247 mm).

Brook Trout were genotyped at 13 microsatellite loci using the methods described by [24]. One individual failed to yield a genotype at multiple microsatellite markers, and was omitted from the remainder of the investigation. Among the remaining individuals, we were able to obtain genotypes at 99.4% of loci (3219/3237 genotypes). Prior to any subsequent analysis, we used COLONY 2.0.5.0 [28] to identify individuals with full-sibs represented in the sample. Where they occurred, full-sibs were randomly removed from the dataset (*n* = 24) until only a single family representative remained in each section. Removal of excess full sibs from the data set reduced the number of multilocus genotypes available within a section by 7.5–11.5% (see S2 Dataset for individual genotypes). Four hundred sixty-nine half sibling pairs were identified among the genotyped individuals, but were left in the data set for analysis.

We used GENALEX, version 6.501 [29], [30], to determine if Brook Trout collected within a stream reach appeared to conform to Hardy-Weinberg equilibrium, based on a Bonferroni-adjusted critical *P*-value (0.0008; α = 0.05). GENALEX was also used to calculate pairwise *F*_ST_ and *G”*_ST_ [31], [32], values among collections in different stream reaches, and conduct analysis of molecular variance (AMOVA) [33]. Next, we used GENEPOP [34], version 4.2.1, to evaluate if there was linkage disequilibrium between loci within our stream reaches. GENEPOP and GENALEX were also used to calculate within-section diversity statistics.

We used STRUCTURE 2.3.4 [35], [36] to determine the most likely number of populations (*K*) present within the study area and examined the results using STRUCTURE HARVESTER [37]. The number of clusters tested ranged from *K* = 1 through *K* = 8 with 20 iterations performed per cluster. Each model run allowed admixture used correlated allele frequencies, and consisted of a burn-in period of 500,000 steps followed by 500,000 iterations that were used for data collection. The models were initially run twice: first without informative priors, and again with the section of capture as an informative prior. Following the hierarchical approach of Vähä et al. [38], we reran STRUCTURE on individual clusters that were identified during the initial runs to check for additional tiers of population structuring. We used CLUMPP 1.1.2 [39] to align repeated model runs and generated ancestry plots using DISTRUCT 1.1 [40].

To quantify long-term rates of genetic exchange among groups inferred using STRUCTURE, we implemented a Bayesian coalescent model in MIGRATE and estimated *θ* and *M*, where *θ* represents the mutation-scaled effective population size (4*N*_e_*μ*) and *M* represents the mutation-scaled immigration rate (*m*/*μ*), where *m* is the proportion of migrants per generation and *μ* is the mutation rate per locus per generation [41], [42]. Migration rates were allowed to be asymmetric and to vary between groups. A preliminary examination of allele frequencies found little support for the stepwise mutation model, so we used the Brownian motion option within MIGRATE and assumed a constant mutation rate across all loci. We used uniform prior distributions for *θ* (0–20) and *M* (0–600). The model ran with an initial burn-in of 50,000 steps, followed by data collection for 200,000 MCMC replicates every 20 steps. Static heating was used (four chains) and the chains were allowed to swap. Modal values from posterior distributions were used for parameter estimates. To calculate long-term genetic exchange rates in units of effective migrants per generation from group *j* to group *i* [43], we used the relationship described by Eq 1 that is independent of the mutation rate. We also examined the skyline plots produced by MIGRATE to determine if the observed genetic differences were the result of recent divergence or historic departure with continued genetic exchange.

$$Immigrants\;per\;generation=\;\frac{\theta_{i}M_{j\to i}}{4}$$ (Eq 1)

### Life History Differentiation

Based on recaptures of individually marked fish, we characterized the physical movement, growth, and survival patterns of Brook Trout within the study area:

#### Movement

We described movement patterns within our study area using mark-recapture observations and stationary PIT antennas. Stationary PIT antennas were operated at two sites within the study area to detect fish movements that would have gone undetected using only a mark-recapture study design (Fig 1; [44]). One antenna array was located on Monroe Run (within section 4) approximately 50 m upstream of its confluence with Big Run. A second antenna array was located at the upstream extent of our study area on Upper Big Run (within section 3). At each location, two pass-by antennas constructed of high-density polyethylene were anchored to the stream bottom and operated between June 2011 and September 2013 [45]. Although battery failures resulted in occasional periods where antennas were not operational, the data derived from these arrays (S3 Dataset) should be a representative sampling of individuals who visited the antenna sites.

#### Growth

We calculated annual growth increments for individuals that were recaptured on consecutive summer electrofishing samples (Eq 2). We used analysis of covariance (ANCOVA) to compare observed growth rates between groups of brook trout while accounting for the effect of individual size for each of the three years of the study. Analysis of covariance models initially considered a size by group interaction term. Where the interaction term was not significant, the models were refitted with only the main effects.

$$Annual\;growth=\frac{L_{1}-L_{0}}{t_{1}-t_{0}}*\;365$$ (Eq 2)

#### Survival

Based on four years of electrofishing surveys, we generated individual encounter histories for 2,973 fish representative of the two streams (1,919 from Big Run and 1,054 from Monroe Run). Forty individuals were captured in both Monroe Run and Big Run during summer sampling events, and were omitted from survival analysis because their genetic association was uncertain. We used multistate Cormack-Jolly-Seber models implemented in program MARK [46] to estimate stage-specific survival rates for Brook Trout. Stages were defined based on total length: young-of-the-year (YOY; <100 mm based on field observations) and adult (A; ≥100 mm). During each annual time step, an individual could survive or die, and all surviving YOY transitioned (Ψ) into adults. Survival rates (*S*) were allowed to vary by year and by stage. Catchability (*p*) was assumed to be constant for each of the summer samples, but modeled independently for fall 2013, as this sample was based on a single pass of electrofishing whereas the other samples used three consecutive passes. We assumed all tags were retained and successfully read during recapture events.

## Results

### Population Genetics

We successfully obtained multilocus genotypes from 249 Brook Trout. Samples from within each of the five sections largely conformed to Hardy-Weinberg equilibrium (Table 1). However, in section 3, two loci showed a departure from Hardy-Weinberg equilibrium (*P* < 0.0038). Significant linkage disequilibrium was detected in one pairwise comparison of loci in section one and in two instances in section 5 (Table 1; *P* < 0.0001).

**Table 1:** Within-section genetic diversity for each of the five study sections calculated using GENALEX and GENEPOP.

Section	*N_Total_*	*N_Analyzed_*	*A*	*H_o_*	*H_e_*	Private alleles	HWE	LD
1. Lower Big Run	40	36	7.692	0.701	0.693	1	13/13	1/78
2. Upper Big Run	52	46	7.462	0.695	0.697	4	13/13	0/78
3. Upper Big Run	53	49	7.769	0.693	0.702	1	11/13	0/78
4. Monroe Run	53	47	7.154	0.670	0.659	1	13/13	0/78
5. Monroe Run	51	47	7.154	0.705	0.685	1	13/13	078

*N*_Total_, sample size before redundant full-sibs were removed from the study; *N*_Analyzed_, sample size after redundant full-sibs were removed from the study; *A*, mean allelic richness; *H*_o_, observed heterozygosity; *H*_e_, expected heterozygosity; HWE, proportion of loci conforming to Hardy-Weinberg equilibrium; LD, the proportion of loci pairs in significant linkage disequilibrium.

**Fig 2:**
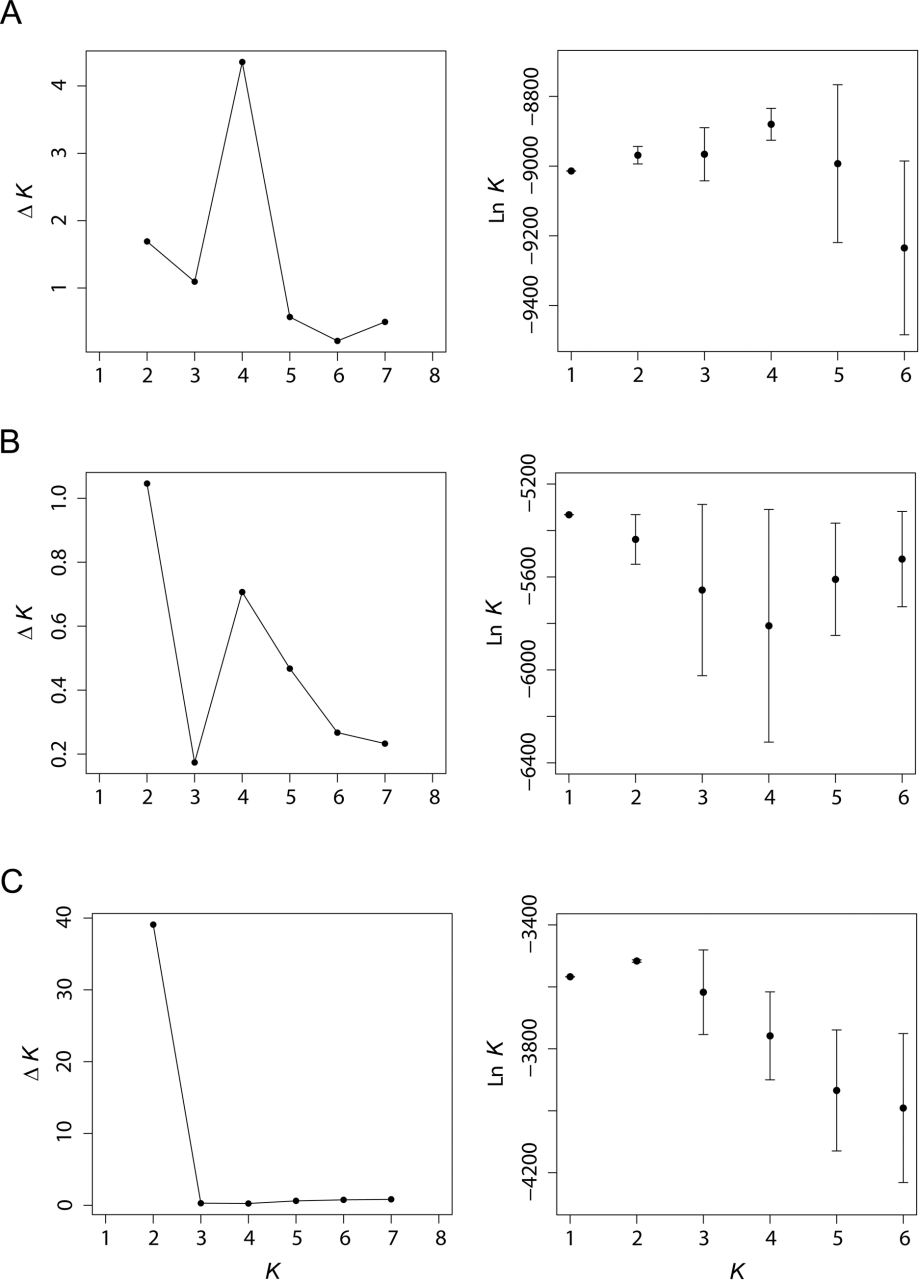
Diagnostic statistics for identifying a most supported *K*. Mean log likelihood (±1 SD) calculated over 20 model runs with informative priors at each *K* and Δ*K*, an *ad hoc* statistic proposed by Evanno et al. (2005) to estimate the correct number of clusters. Likelihoods for *K* 7 through 8 were omitted for graphical purposes, but were less than the values shown for *K* 1 through 6. Panel A was derived from a global analysis that considered all 225 individual across the five study sections. Panel B reflects a nested analysis that examined only those individuals collected in Big Run (sections 1–3). Panel C was derived from a nested analysis that focused on fish collected in Monroe Run (sections 4–5).

**Fig 3:**
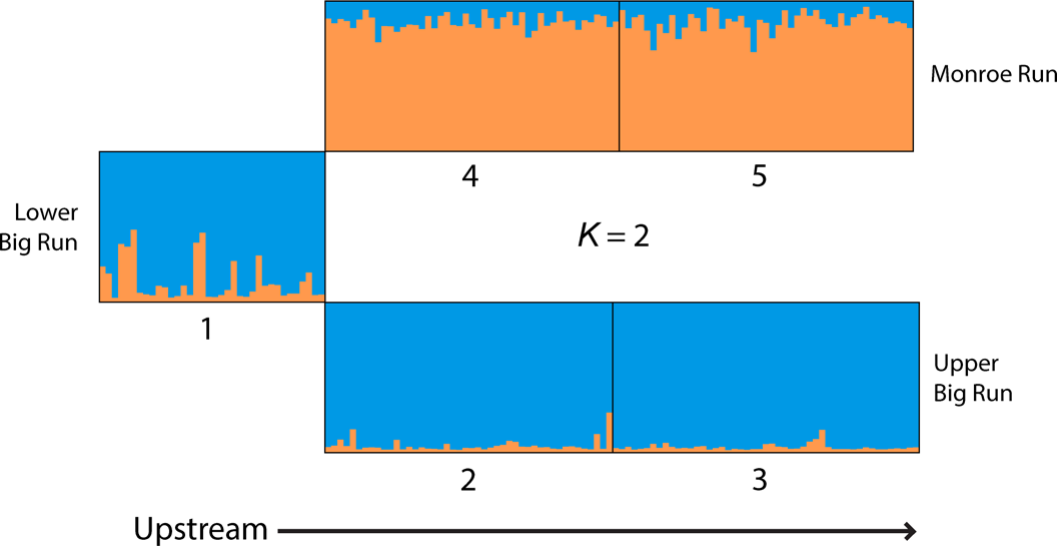
Individual assignment probabilities across five stream sections based on two clusters (*K* = 2). Stream sections were considered as prior information and correlated allele frequencies were used.

Hierarchical Bayesian genetic clustering showed support for at least two genetic groups that inhabit the study area, based on comparisons of Delta *K* and model likelihoods for models considering *K* = 1 through *K* = 8 (Program STRUCTURE; Fig 2A), regardless of whether or not informative priors were used. Inferred membership of individuals in the two genetic clusters clearly reflected the geographic layout of the stream network, with Monroe Run fish consistently assigned to one group while Big Run fish upstream of the confluence with Monroe Run were consistently assigned to another (Fig 3). Genetic differentiation was small, but significant between streams (*F*_ST_ = 0.011–0.013 and *G”*_ST_ 0.029–0.047; Tables 2 and 3), and no differentiation was detected among reaches within streams. Overall, 1.22% of the observed molecular variance could be attributed to differences between upper tributaries (AMOVA). Downstream of the confluence of Monroe and Big Run, some fish showed signs of intermediate ancestry (e.g., *Q* = 0.46 and *Q* = 0.48). However, the majority of individuals in Lower Big Run clustered strongly with those in Upper Big Run, and none of these individuals grouped with the fish from Monroe Run. Upstream of the confluence, all individuals in the tributaries showed low (but often non-zero) values of ancestry in the non-resident cluster, suggesting persistent but low levels of gene flow between groups (Fig 3).

**Table 2:** Pairwise *F*_ST_ values among Brook Trout collected in five study sections in the Big Run watershed.

Section number and location	Section number				
	1	2	3	4	5
1. Lower Big Run	-	0.423	0.881	**0.005**	**0.004**
2. Upper Big Run	0.006	-	0.386	**0.002**	**0.001**
3. Upper Big Run	0.005	0.006	-	**0.001**	**0.001**
4. Monroe Run	0.011	0.012	0.013	-	0.293
5. Monroe Run	0.011	0.012	0.011	0.006	-

*F*_ST_ values are shown below the diagonal and *P*-values are shown above. Values that were statistically significant (based on a Bonferroni-adjusted critical *P*-value of 0.005) are shown in bold.

**Table 3:** Genetic divergence among Brook Trout collected in five study sections in the Big Run watershed.

Section number and location	Section number				
	1	2	3	4	5	
1. Lower Big Run	-	0.414	0.886	**0.003**	**0.003**
2. Upper Big Run	0.001	-	0.376	**0.000**	**0.000**
3. Upper Big Run	0.000	0.002	-	**0.000**	**0.000**
4. Monroe Run	0.031	0.041	0.049	-	0.316
5. Monroe Run	0.031	0.041	0.037	0.003	-

Pairwise *G”*_ST_ values are shown below the diagonal and *P*-values are shown above. Values that were statistically significant (based on a Bonferroni-adjusted critical *P*-value of 0.005) are shown in bold.

Additional modeling within STRUCTURE found no support for additional population structuring within Big Run (sections 1–3; Fig 2B). However, there was evidence to suggest that Monroe Run (sections 4–5; Fig 2C) may harbor an additional tier of genetic structuring, with two further subgroups supported. These two subgroups showed no obvious sorting within Monroe Run, and the majority of fish (89/94) clustered together. For the purposes of this manuscript, we chose to focus our remaining analyses on contrasting fish in Big Run with those in Monroe Run, reflecting the highest level of genetic structure observed.

The long-term rate of genetic exchange between the two groups was low, given the close geographic proximity of each group (3.57 effective immigrants per generation from Monroe Run into Big Run and 6.28 effective immigrants per generation from Big Run into Monroe Run; Table 4). Assuming a three year generation time (D. Kazyak, unpublished data), annual immigration is 1.19 effective migrants into Big Run and 2.09 effective migrants into Monroe Run. Based on the skyline plots, genetic exchange was historically low between the streams but has increased in recent generations (S1 Fig).

**Table 4:** Estimates of key parameters derived from the coalescent using MIGRATE.

Population	θ_*i*_	*Μ_j->i_*	Effective immigrants/generation	Effective immigrants/year*
Big Run (Sections 2–3)	1.17 (0.60–1.67)	12.20 (1.20–24.00)	3.57 (0.18–10.02)	1.19 (0.06–3.34)
Monroe Run (Sections 4–5)	0.59 (0.15–1.05)	42.60 (24.00–81.60)	6.28 (0.90–21.42)	2.09 (0.30–7.14)

θ, mutation-scaled effective population size; *M*, mutation-scaled immigration rate. Values in parentheses represent the 95% probability interval derived from the posterior distribution.

*Based on an average generation time of three years.

### Life History Differentiation

Based on evidence for two genetically distinct groups occupying adjacent streams, subsequent life history trait analyses were applied separately to Big Run (sections 1–3) and Monroe Run (sections 4–5) Brook Trout.

#### Movement

Exchange of individuals between the two streams was rare based on four years of electrofishing survey data. Brook Trout initially tagged in Monroe Run (1,058 individuals) were never captured during electrofishing surveys in Big Run above the confluence (Table 5). Furthermore, trout tagged in upper Big Run (1,443 individuals) were very rarely detected in Monroe Run (*n* = 4), and three of the four detections were within 50 m of the confluence. Among fish tagged in either stream, 99.2% of relocations occurred within the same stream, and 89.4% of relocations were within the same section where an individual was tagged. When a fish left the stream of initial capture, it was found below the confluence in 12/16 instances (Table 5). Furthermore, based on recaptures during consecutive fall sampling events, very few fish moved between the tributaries from year to year (Table 6). To summarize, exchange of Brook Trout between the two streams was rare based on the electrofishing survey data.

**Table 5:** Distribution of relocations of Brook Trout initially tagged during the summer in five contiguous stream reaches based on physical recaptures and stationary passive integrated transponder antennas.

			Number of physical recaptures					Number of individuals detected	
Section	Description	*n*	1	2	3	4	5	Big Run Array	Monroe Run Array
1	Lower Big Run	513	277	46	6	14	3	30	42
2	Upper Big Run	686	4	514	64	3	0	75	6
3		757	0	35	486	1	0	182	2
4	Monroe Run	447	7	0	0	390	69	3	60
5		611	1	0	0	8	523	1	16

The number of fish tagged in each reach is denoted by *n*.

**Table 6:** Estimates of migration rates between Big Run and Monroe Run based on mark-recapture data for individuals collected in consecutive fall sampling events and summer population estimates.

Population	*N*	Emigration (%)	Immigrants/year
Big Run (Sections 2–3)	687	0.70%	9.13
Monroe Run (Sections 4–5)	377.25	2.42%	4.77

The average observed abundance (2010–2013) is represented by *N*.

Movement patterns recorded at the two stationary antennas were similar to those inferred from our mark-recapture observations. The stationary PIT antenna arrays recorded >160,000 tag detections representing 413 of 3014 (13.7%) of the tagged individuals. Most of the detections were due to a few individuals that were repeatedly detected. The antennas at the downstream terminus of Monroe Run recorded 126 unique fish that comprised primarily individuals tagged in Monroe Run (*n* = 76), and to a lesser extent, individuals that were tagged in Lower Big Run (*n* = 42). Fish that were initially tagged in Upper Big Run were very seldom recorded on the Monroe Run antennas (*n* = 8). The antennas at the upper extent of the study area on Upper Big Run recorded 291 unique Brook Trout. These individuals were mostly tagged on Upper Big Run (*n* = 257) and Lower Big Run (*n* = 30), with only a few trout originating in Monroe Run (*n* = 4). The majority of tag detections at both arrays represented individuals that had been tagged in sections adjacent to the stationary antennas (68.1%). Overall, the observed movement patterns suggest a large degree of isolation between the two streams. When exchange occurred, it was generally individuals transitioning between Lower Big Run and one of the tributaries, rather than between the two tributaries.

#### Growth

**Fig 4:**
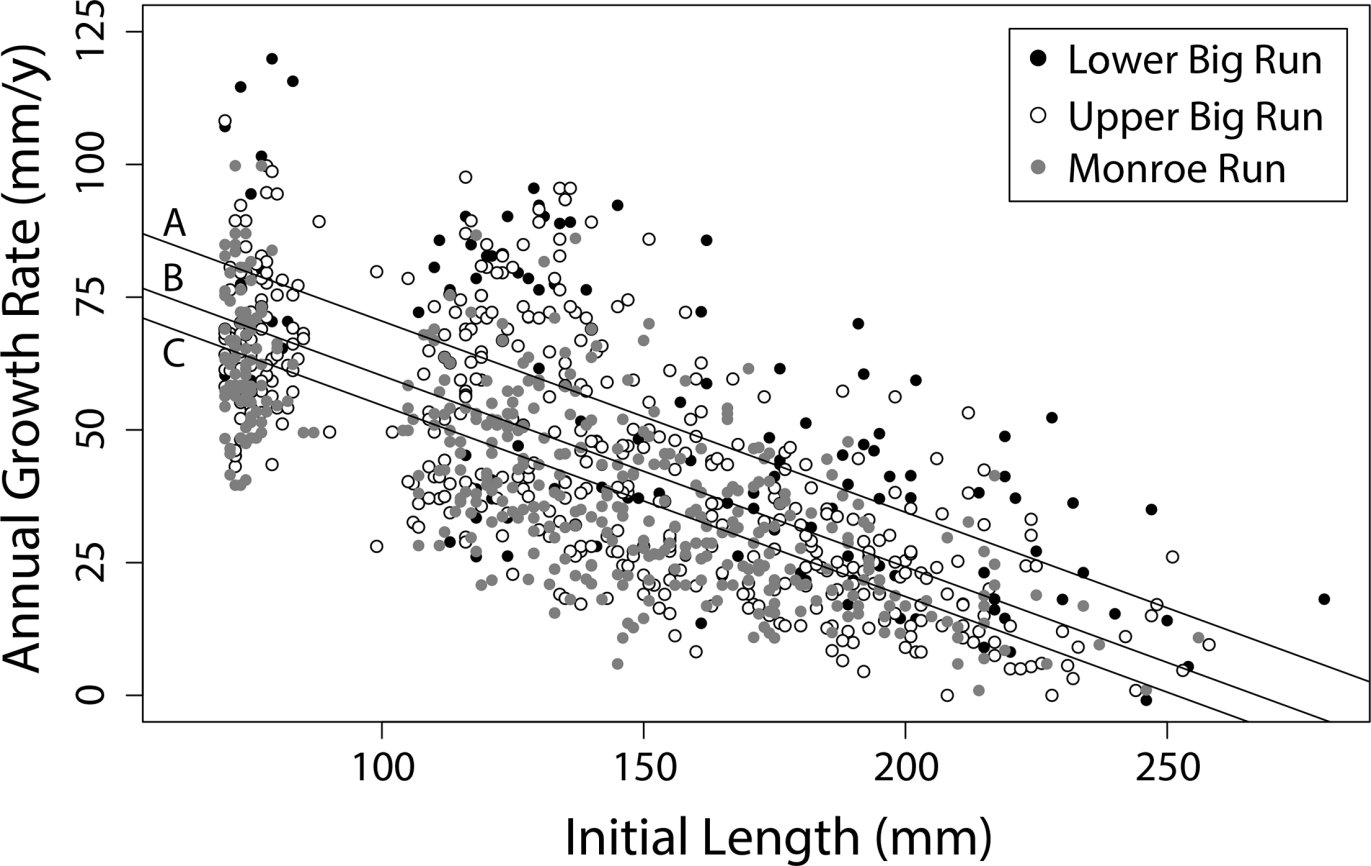
Observed annual growth rates for Brook Trout in Big Run and Monroe Run, 2010–2013. The regression lines represent mean growth rate at length for Lower Big Run (A), Upper Big Run (B), and Monroe Run (C).

Annual growth increments ranged from 0 to 119.9 mm, and decreased linearly with respect to body size in each year (*P <* 0.05). Despite substantial interannual variation, we found significant differences in growth rates (ANCOVA; *P* < 0.05) between individuals in Big Run and Monroe Run for each of the three years of record. Brook Trout in Big Run (sections 1–3) consistently grew faster (overall 7.8 mm/y faster at a given length; *P* < 0.05) than in Monroe Run (sections 4–5; Fig 4). Subsequent analysis found that this difference was primarily driven by rapid growth in Lower Big Run (section 1), where growth was significantly faster than Upper Big Run (sections 2–3; mean = 10.3 mm/y faster at a given length; *P* < 0.05) and Monroe Run (sections 4–5; mean = 15.9 mm/y faster at a given length; *P* < 0.05). Brook Trout in Upper Big Run grew significantly faster than conspecifics in Monroe Run, but the effect size was small (5.6 mm/y faster at a given length; *P* < 0.05). Overall, stream reach accounted for 4.8% of the observed variation in growth rates.

#### Survival

**Fig 5:**
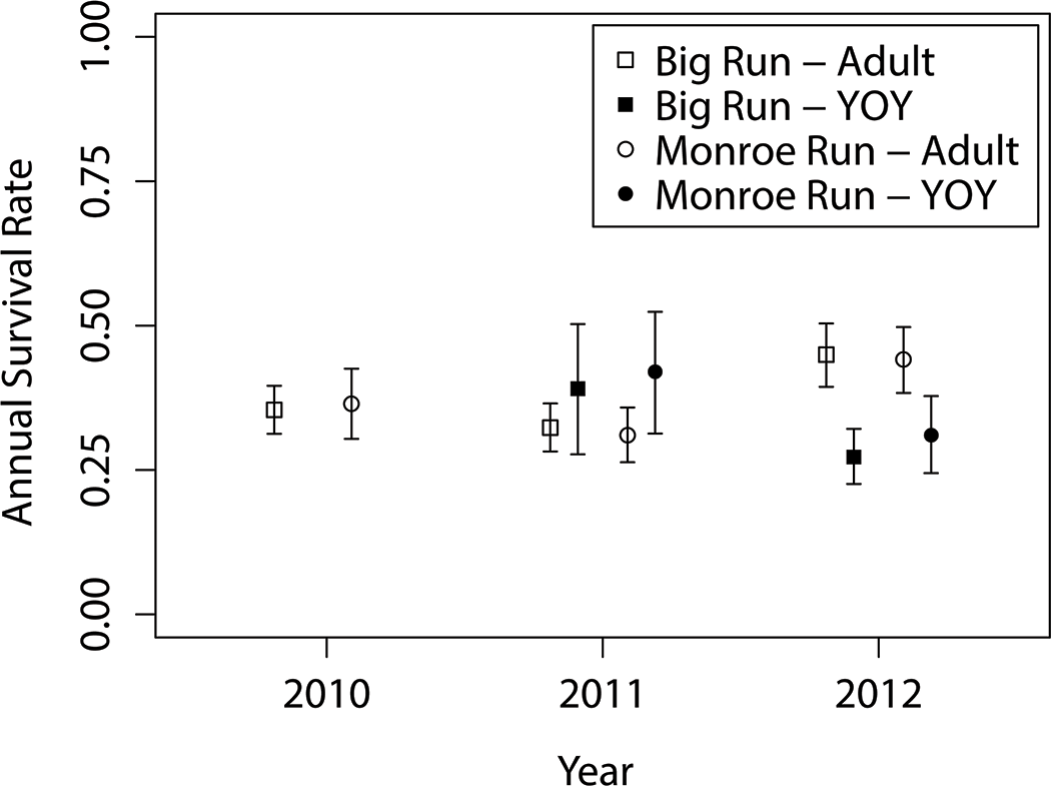
Survival estimates derived from multistate Cormack-Jolly-Seber models fit to fish in Big Run and Monroe Run. There were insufficient numbers of young-of-the-year (YOY) fish collected in 2010 to generate meaningful estimates of survival rates.

Estimated annual survival rates ranged from 31.0–45.0% for adults, with no significant difference in year-specific survival rates between the streams (Fig 5). Similarly, young-of-the-year survival rates ranged from 27.2–42.0% and were similar among the streams in 2011 and 2012 (Fig 5). Unfortunately, low recruitment in 2010 prohibited meaningful estimates of young-of-the-year survival rates for both streams.

## Discussion

The Brook Trout within the Big Run watershed form at least two genetically distinct groups inhabiting adjacent and physically connected streams, with no obvious barriers to dispersal. The physical movement patterns exhibited by these groups appear to limit the potential for genetic exchange between streams, reinforcing the observed genetic structure. When integrated over the evolutionary history of these populations, the estimated long-term rates of genetic exchange between the two streams show a small but non-zero degree of connectivity. Thus, exchange occurs between streams, but introgression rates are typically limited to a few immigrants per generation.

We believe the Brook Trout in this study system represent a cryptic metapopulation: a spatially continuous distribution of organisms exhibiting metapopulation-like behaviors. Although Levins [47], [48], focused on extinction-recolonization dynamics in his original model of the metapopulation concept, later interpretations relaxed the definition to include groups of geographically and/or genetically structured local subpopulations that remain connected by migration but at rates that are not sufficient to homogenize the entire group [49], [50]. Our study presents evidence of the latter scenario in lotic Brook Trout at a very local spatial scale and in the absence of physical barriers to migration. Despite decades of population monitoring, the fine-scale genetic divergence and metapopulation-like structure of Brook Trout within the Big Run watershed remained unnoticed. Moreover, genetics sampling efforts conducted at a single point location or across a single tributary would likely fail to detect any population structure and overlook important reservoirs of biodiversity. Our study examined <20% of the Big Run stream network, so it is possible that additional population structuring occurs even within our small study watershed.

Increasingly, population structure has been revealed in many groups of organisms that were previously thought to represent single populations [51–53]. In many cases, these organisms have a high dispersal capability or live in continuous habitat, leading to an expectation of panmixia [54]. In streams, where dispersal is often limited and habitats are highly heterogeneous, we should expect population differentiation to be even more prevalent. Although some migration barriers such as waterfalls or culverts are readily identified, other factors that influence dispersal and genetic exchange may be less obvious [55]. Water chemistry and temperature, both of which vary continuously through time and space, may restrict or prohibit genetic exchange within stream networks based on physiological limits of the organisms [25]. If such isolation by ecological resistance is commonplace, then cryptic metapopulations may be widespread, yet have largely remained undetected.

The existence of cryptic metapopulations has implications for the ecology of stream fishes. Genetic population structure may serve to enhance resiliency by stabilizing abundance through time, as in the context of a portfolio effect [7]. Undetected structuring within populations may also help explain the observed life history variation in many species [56], [57]. The presence of cryptic metapopulations may increase standing genetic diversity that can facilitate future adaptation [58]. Thus, cryptic metapopulations may influence both the contemporary population dynamics and future adaptive potential of stream fishes.

Within a stream network, patterns of local selection and limited connectivity may lead to population differentiation in traits. Among salmonids, local adaptation is relatively common, sometimes evident on small spatial scales (a few kilometers), and can develop rapidly (<20) generations; [59], [60]. Local adaptation may be manifest in many traits, including morphology [3], thermal response [61], movement patterns [18], and vital rates [18], [62]. We did not observe strong, consistent differences in vital rates between the two populations. Although a coarse-scale population comparison found growth rates to be faster in the Big Run population, further analysis revealed that this difference was largely attributable to enhanced growth in Lower Big Run relative to the tributaries. Upper Big Run and Monroe Run offer similar habitats (Table 7), and the observed differences in growth rates were small. In contrast, Brook Trout in Lower Big Run grew significantly faster. Lower Big Run is generally wider and offers more extensive pool habitat (Table 7). While the apparent productivity of this downstream habitat may drive the observed movement patterns between Lower Big Run and upstream areas [63], it is unlikely to cause the observed differentiation among populations. Both populations can freely move between their host tributary and productive downstream areas. Additionally, stage-specific survival rates were very similar for the two streams in each year. Thus, we lack compelling evidence to conclude that environmental differences between the streams are generating discrepancies in vital rates and different selective pressures. If natural selection is driving differentiation between these populations, the mechanisms remain unclear.

**Table 7:** Comparison of key habitat metrics among Lower Big Run, Upper Big Run, and Monroe Run. Maximum depth, residual pool depth, and pool coverage are presented as an average value for a 50 m reach ± 1SD. Temperature data were derived from loggers deployed at fixed sites within the study area from summer 2012 through spring 2014 and are reported as a mean ± 1SD. Water chemistry data was collected by the Maryland Biological Stream Survey in 1996 (Big Run; GA-A-090-310-96) and 2000 (Monroe Run; SAVA-101-C-2000). Water chemistry data was not available (NA) for Lower Big Run.

Habitat metric	Lower Big Run	Upper Big Run	Monroe Run
	(1)	(2–3)	(4–5)
Total length (km)	0.95	2	1.5
Wetted width (m)	6.0 ± 2.0	4.4 ± 1.5	3.6 ± 1.2
Maximum depth (cm)	60.2 ± 36.0	39.1 ± 18.4	34.9 ± 12.3
Residual pool depth (cm)	42.9 ± 32.3	27.9 ± 18.3	23.5 ± 11.8
Pool (%)	18.9 ± 14.9	12.8 ± 11.3	11.7 ± 11.8
*Mean seasonal temperature* (°C)			
Winter	1.9 ± 1.9	2.2 ± 1.8	1.8 ± 1.8
Spring	9.9 ± 4.1	9.6 ± 3.8	9.9 ± 4.1
Summer	17.0 ± 1.9	16.5 ± 2.0	16.9 ± 2.1
Fall	7.2 ± 4.3	6.9 ± 4.1	6.7 ± 4.3
*Maximum temperature* (°C)			
2012	21.6	21.7	21.9
2013	20.5	19.2	20.4
*Water chemistry*			
pH	NA	7.06	7.15
Conductivity (μS/cm)	NA	50	70
Nitrate (ppm)	NA	0.5	0.3
Sulfate (ppm)	NA	11.8	12.3
Dissolved organic carbon (ppm)	NA	0.9	1.1

Tributary confluences are often pronounced zones of ecological change [12], [64], [65], and have sometimes been associated with genetic boundaries [17], [66] or clines [16]. The tributary-specific movement patterns we observed may promote reproductive isolation and help maintain the observed population structure. Despite the absence of barriers to movement within the study area and the potential mobility of Brook Trout, individuals very seldom moved between the two tributary reaches (Table 6) and we observed limited movement overall, even between adjacent reaches within the same tributary. We currently lack evidence to know if individuals that moved into Lower Big Run returned to their home tributaries to spawn, but we speculate that little genetic exchange occurs among the two groups of Brook Trout where they co-occur in Lower Big Run. Based on fall spawning surveys, reproductive effort is minimal in Lower Big Run relative to its tributaries. This lack of spawning activity may enhance the genetic separation between tributaries, since this is the only area where we found much spatial overlap between the genetic groups. Positive assortative mating may be a further source of genetic isolation. In a stream where three strains of Brook Trout were stocked in sympatry, Richards et al. [26] found the majority of reproduction arose from same-strain matings. Thus, mechanisms for isolation exist among sympatric Brook Trout strains, either due to behavioral isolation or reduced fitness among admixed offspring, and this might help to explain the large degree of genetic population structure found throughout its native range ([25], King unpublished data). An alternative behavioral mechanism for isolation may be that natal homing limits straying among tributaries during the spawning season, thus moderating the effects of seasonal dispersal. We speculate that Brook Trout may use olfactory cues to guide movement patterns and reproductive behavior, ultimately contributing to reproductive isolation. This behavior is well-documented in salmonids [20], [67–69], and may be broadly conserved across freshwater fishes [70], [71].

Although new analyses may show cryptic metapopulations to be widespread in streams, it may be unrealistic to manage such taxa. The scale and scope of the effort required to identify and individually manage these population components is simply not feasible in most cases, particularly if they are not spatially segregated. However, it is important to recognize and conserve the underlying population structure, as the subpopulations may represent local adaptation and reservoirs of genetic diversity. When fishes are extirpated from a stream reach within a larger watershed, genetically distinct populations may be wiped out, even if other conspecifics later recolonize the habitat. Additionally, restoration and reintroduction activities may be more successful if the population structure is understood, especially when dealing with species known for high amounts of genetic divergence such as Brook Trout. For example, a dwindling population may be more difficult to restore by improved connectivity or manual transfers [72] if fishes exhibit population divergence on a small geographic scale because the underlying mechanism for divergence may inhibit interbreeding [73]. Further, efforts to improve connectivity may be overstated if fish populations have naturally evolved to persist in small patches, even where larger areas of interconnected habitat are available. When genetics studies are conducted, special attention should be placed on the sampling scheme, as study design can influence our inferences about population structure [74].

Cryptic metapopulations may play an important role in the ecology of stream fishes, but are easily overlooked where organisms are continuously distributed. Future work should seek to determine the prevalence of cryptic metapopulations and seek to understand their roles in stream ecology. An improved understanding of this hidden level of population organization and the mechanisms that maintain it may help to improve management and restoration outcomes.

## Supporting Information

S1 Dataset Physical captures of tagged individuals. (CSV)

S2 Dataset Microsatellite genotypes. (CSV)

S3 Dataset Stationary PIT antenna detections. (CSV)

S1 Fig Summary of parameter values over time (across all loci) generated by MIGRATE for Brook Trout in Big Run (1) and Monroe Run (2). Time is scaled by the mutation rate per generation, which is not well-documented for microsatellites in Brook Trout. The gray bar represents the 95% confidence interval. Red indicates areas where the 95% confidence interval exceeded the bounds of the figure. The shaded bar above each panel refelects the amount of data used to calculate each value. Parameter estimates in areas with darker shades were based on a larger number of samples per bin.
(TIF)
