# Supplementary figures and images for: Hiding in Plain Sight: A Case for Cryptic Metapopulations in Brook Trout (Salvelinus fontinalis)
Source: PLoS One. 2016 Jan 5;11(1):e0146295. doi: 10.1371/journal.pone.0146295 (PMC4701135; doi:10.1371/journal.pone.0146295)

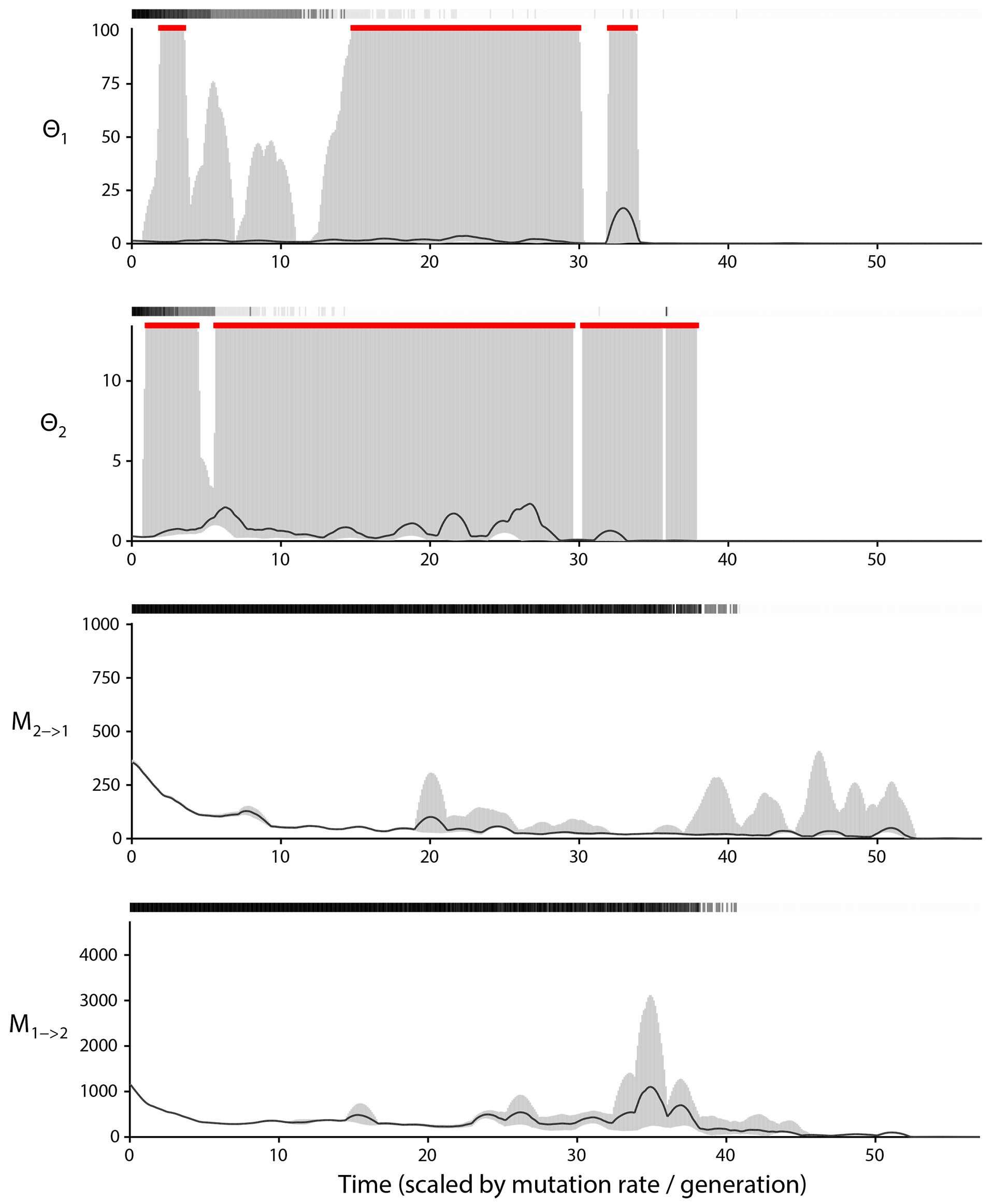

Supplement: S1 Fig — Time is scaled by the mutation rate per generation, which is not well-documented for microsatellites in Brook Trout. The gray bar represents the 95% confidence interval. Red indicates areas where the 95% confidence interval exceeded the bounds of the figure. The shaded bar above each panel refelects the amount of data used to calculate each value. Parameter estimates in areas with darker shades were based on a larger number of samples per bin. (TIF) [file pone.0146295.s004.tif]
